# Performance of Children with and without Auditory Processing Disorders in Adaptive Temporal Gap Detection Measures

**DOI:** 10.1055/s-0045-1804517

**Published:** 2025-10-09

**Authors:** Chandni Jain, Kishore Tanniru, Gayathri Kalarikkal

**Affiliations:** 1 Department of Audiology, All India Institute of Speech and Hearing, Manasagangothri, Mysuru, Karnataka, India; 2 Department of Rehabilitation Sciences, College of Applied Medical Sciences, King Saud University, Riyadh, Kingdom of Saudi Arabia

**Keywords:** auditory processing disorders, gap detection, temporal resolution, adaptive threshold procedures, across-channel temporal gap detection

## Abstract

**Introduction:**

Children with auditory processing disorder (APD) exhibit various auditory processing deficits, including temporal processing deficits. Temporal processing abilities are assessed by estimating the gap detection threshold (GDT) as the lowest perceivable gap duration identified by the subject.

**Objective:**

The present study attempted to examine the performance of normal-hearing children and children with APD using adaptive within-channel and across-channel gap detection tests.

**Methods:**

Two groups of children aged between 10 and 12 years participated in the study. Group 1 included children diagnosed with APD, and group 2 included normal hearing, typically developing children (TD), with 12 participants in each group. For each subject, the lowest detectable gap duration was obtained monoaurally, using broadband noise (BBN), within-channel (narrow bands of noise centered spectrally at 2 kHz on either side of the gap), and across-channel (narrow bands of noise leading marker spectrally centered at 2 kHz and trailer marker spectrally centered at 1 kHz) gap detection tests through the Psycon platform (free).

**Results:**

The results of the statistical analysis revealed significant group differences only in across-channel GDT measures between the two groups. In contrast, there were no statistically significant differences between the groups in terms of either within-channel GDT or BBN GDT.

**Conclusion:**

The results indicate that, compared to other stimuli, an across-channel gap detection test would be a better diagnostic test of temporal resolution to identify and assess children with APD.

## Introduction

Auditory temporal gap detection is an aspect of auditory processing that enables individuals to perceive silent intervals between two successive auditory stimuli. Accurate processing of auditory temporal gap ability is crucial in various auditory tasks, such as speech perception, 1 2 music appreciation, 3 and sound location. Presently, two different methods of gap detection assessment measures are explored to evaluate temporal gap processing difficulty. These methods are broadly categorized into within-channel gap detection threshold (WGDT) and across-channel gap detection threshold (AGDT) tests. 4 In WGDT, the stimuli before and after the silence are within the same auditory spectrum (broadband/white noise or frequency-specific narrow-band noise). In contrast, in AGDT, the stimuli differ spectrally in center frequencies before and after the silence. Within-channel temporal processing requires the involvement of a preassumed single neural channel, whereas across-channel processing requires comparative timing between the two different auditory neural channels. 5 The lowest perceived silence intervals estimated on AGDT are often reported to be higher (indicating poorer performance) than those on WGDT in individuals with normal hearing sensitivity. 4 6 7 Further, AGDT measures have also been reported to be highly dependent on the frequency separation among the markers. 8 9 Poor performance in AGDT tasks is attributed to the complexity of the task involved.

Overall, gap detection threshold (GDT) measures have proven to be efficient, and they constitute an important part of the diagnostic tools to differentiate individuals with auditory processing disorders (APDs) from their peer group with normal hearing abilities. 10 Furthermore, children with APD often exhibit various auditory processing deficits, which include abilities related to temporal processing. 11 12 Dawes et al. 13 examined temporal processing abilities in children diagnosed with central APD using gaps in noise (GIN) and random GDT (RGDT) for various gaps embedded in pure-tone signals and other APD test batteries. The study 13 highlighted the importance of assessing temporal processing deficits in children with APD and nonsignificant classification of etiological factors among APD. Even though more attention is directed toward estimating GDT using broadband noise (BBN), Phillips et al. 14 assessed GDT in normal children and children referred due to central APD using WGDT and AGDT measures with broad spectral noise markers. The study 14 showed that WGDT was similar between the two groups, while AGDT varied significantly. This indicates that AGDT tasks are more sensitive to the perceptual disturbances in APD than WGDT tasks.

Apart from the variations in the stimuli, Hoover et al. 15 noted significant differences in the comparison of estimated GDT using clinical and traditional procedures, especially in older adults. The primary differences among these procedures lie in their adaptive nature and stimulus presentation. With advances in clinical-testing technology, the traditional procedures thought to be complex are gaining more attention and becoming more clinical with the assistance of advanced computing technology. Such procedures offer distinct approaches to determine the threshold at which an individual can reliably detect temporal gaps, each with advantages and limitations. Adaptive procedures such as the staircase or maximum-likelihood procedure modify the gap duration after each trial based on the participant's response and make subsequent trials more challenging or easier. Such an approach efficiently converges on the participant's threshold by tailoring the stimulus parameters to individual sensitivity, reducing the number of trials needed for threshold estimation and minimizing participant fatigue. 16 In audiological test procedures, adaptive procedures have also been proven reliable in testing younger-age children. 17

Evidence of deviated performance in different variations of GDT measures in children diagnosed with APD is highly limited. Regarding perceptual GDT measures, only one study, by Phillips et al., 14 reported that AGDT measures are significantly more deviant in children at risk of developing APD than in their peers. However, the study 14 did not involve any precise adaptive procedures of GDT estimation and provided only the best gap durations instead of the absolute GDT. Furthermore, the GDT measures were performed in children, including those at risk for APD, who had not received a confirmed diagnosis. Thus, to fill the scarcity of evidence-based research comparing within- and across-channel gap detection abilities using adaptive procedures, especially in children diagnosed with APD, the present study attempted to examine the performance of children diagnosed with APD along with their peer group with normal hearing in within- and across-channel gap detection measures using adaptive staircase procedures.

## Methods

Two groups of children aged between 10 and 12 years participated in the study. Group 1 included children diagnosed with APD, and group 2 included typically developing children (TD) with normal hearing, with 12 participants in each group. All participants exhibited pure tone averages lower than 15 dB HL across octave frequencies ranging from 250 Hz to 8,000 Hz for air conduction, and from 250 Hz to 4,000 Hz for bone conduction. Their speech identification scores ranged from 80% to 100% in both ears in quiet, and immittance evaluation indicated normal middle-ear functioning. None of the children reported neurological, otological, vestibular, or speech-language issues.

The TD group was composed of children who successfully passed the Screening Checklist for Auditory Processing in Children (SCAP) 18 and the Screening Test for Auditory Processing (STAP). 19 However, along with the SCAP and STAP, a series of tests were performed, such as the dichotic consonant-vowel test, 20 the duration pattern test, 21 the speech perception in noise (Indian English), 22 the revised auditory memory and sequencing test in Indian English, 23 and the GIN test to identify children with APD. Children who failed any two tests by one standard deviation (SD) of normative data or any one of the tests by two SDs of normative values in the test battery were diagnosed with APD and considered for the present study upon consent from the parents. Individual auditory processing test profiles of the subjects with APD are presented in Table 1

**Table 1:** Individual auditory processing test profiles of children in the auditory processing disorder group

No.	Age	Sex	*SPIN*		*RAMST-IE*	*DCV*			*DPT*		*GDT*	
			R	L		R	L	DC	R	L	R	L
Subject 1	12	M										
Subject 2	12	M										
Subject 3	12	F										
Subject 4	11	F										
Subject 5	11	F										
Subject 6	11	F										
Subject 7	10	F										
Subject 8	10	M										
Subject 9	11	M										
Subject 10	10	M										
Subject 11	10	F										
Subject 12	12	M										

**Abbreviations:** DC, double correct scores; DCV, dichotic consonant-vowel test; DPT, duration pattern test; F, female; GDT, gap detection test; L, left ear score; M, male; R, right ear score; RAMST-IE, revised auditory memory and sequencing test; SPIN, speech perception in noise.

**Note:** The shaded fields indicate failure in that particular test.

### Test Environment

The test was performed in a quiet classroom with minimal auditory or visual distractions. 24 The test room was chosen to be located away from the school and high-traffic areas, including the classroom, playground, canteen, and generator room. The test was conducted on a personal laptop using HD 569 headphones (Sennheiser Electronic GmbH & Co. KG, Wedemark, Hanover, Germany) calibrated to 65 dB SPL.

### Stimuli and Procedure

For each subject, the lowest detectable gap duration was obtained using BBN, within-channel (narrow bands of noise centered spectrally at 2 kHz on either side of the gap) and across-channel (narrow bands of noise leading marker spectrally centered at 2 kHz and trailer marker spectrally centered at 1 kHz) gap detection tests through the Psycon platform (free). The order of presentation of the stimuli for each participant was randomized. Gaussian noise devoid of any edge filters was promptly generated via code, featuring a ramp duration of 10 ms at both onset and offset phases. This served as the standard stimulus to assessing GDT through BBN. An adaptable symmetrical gap, ensuring silence, was introduced around the midpoint of the signal, approximately 250 ms into the signal's duration, thus preserving a total signal duration of 500 ms within the adjustable stimuli.

Further, WGDT was quantified by employing identical leading and trailing markers derived from filtered narrow-band noise. These markers were promptly generated from Gaussian noise possessing a 1/4th octave bandwidth and a frequency geometrically centered at 2 kHz. The use of an 8th-order Butterworth filter, characterized by a passband ripple of 0.5 dB and stopband attenuation of −40 dB, facilitated the extraction of precise markers. Conversely, to assess the AGDT, the leading marker was centered at 2 kHz, while the trailing marker comprised 1 kHz centered narrow-band noise. In measuring the WGDT and AGDT, the standard stimulus was also equipped with a 1-ms ramp around a 1-ms gap to eliminate the potential influence of transition cues on the GDT assessment. Moreover, the duration of the trailing marker varied between 250 and 350 ms per trial, mitigating the impact of durational cues during the GDT evaluation.

An adaptive staircase method, employing 2 alternative forced-choice paradigms with a 2-down 1-up procedure, was utilized to present any of the stimulus. The initial 8 reversals employed a step-size factor of 1.25, subsequently adjusted to a factor of 1.05 for the final 4 reversals. Detailed insights into the stimulus design and methodology can be obtained from Alhaidary et al. 6 or Alhaidary and Tanniru. 25 An average of the final 4 reversals was computed as the measured GDT, and their standard deviations were noted. An inter-stimulus interval of 500 ms and an inter-trial interval of 1,000 ms were used while presenting the stimulus for all subjects.

### Analysis

The collected data was entered into the IBM SPSS Statistics for Windows (IBM Corp., Armonk, NY, United States) software, version 25.0. The raw data was subjected to a normality test. A standard group comparison design was used to compare the gap detection abilities in children with and without APD.

## Results

The GDTs using BBN, WGDT, and AGDT tests were compared in children with and without APD. The data obtained was tested for normality using the Shapiro-Wilk's test of normality. The result showed that the data presented non-normal distribution ( *p* < 0.05) for the APD group, except for the AGDT score in the left ear ( *p* = 0.169), and normal data distribution ( *p* > 0.05) for the TD group, except for the WGDT score in the right ear ( *p* = 0.037). The mean, median, SD, and range values of all the measures for the right and left ears are shown in Table 2 .

**Table 2:** Results of the gap detection measures for the study sample

Task (ear)	Mean		Median		Range				SD	
					Minimum		Maximum			
	TD	APD	TD	APD	TD	APD	TD	APD	TD	APD
BBN GDT (right ear)	2.1	5.6	2.3	2.8	0.8	0.8	2.8	28.3	0.6	7.8
BBN GDT (left ear)	2.3	4.9	2	2.5	1.5	1.0	3.3	17.0	0.5	5.7
WGDT (right ear)	13.4	34.8	15.3	8.3	3.0	2.0	21.0	132.5	7.3	49.7
WGDT (left ear)	10.1	34.4	9	22.1	2.5	1.0	21.0	112.0	6.4	38.3
AGDT (right ear)	48.7	94.7	43.8	83.0	12.0	53.5	92.0	215.5	21.4	41.9
AGDT (left ear)	54.8	101.5	53	104.5	13.0	67.0	97.5	134.5	22.8	24.6

Abbreviations: AGDT, across-channel gap detection threshold; APD, auditory processing disorder; BBN, broadband noise; GDT, gap detection threshold; SD, standard deviation; TD, typically-developing; WGDT, within-channel gap detection threshold.

**Fig. 1:**
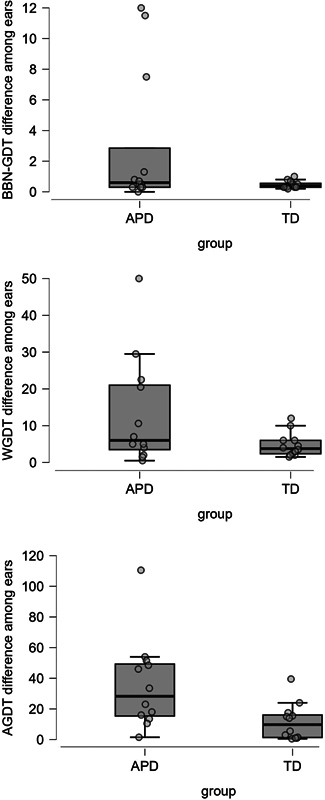
Scatterplot distribution with corresponding box plots for absolute individualized ear differences on GDTs obtained with various stimuli for TD and APD children. **Abbreviations:** AGDT, across-channel (2–1 kHz) gap detection threshold; APD, auditory processing disorder; BBN, broadband noise; GDT, gap detection threshold; TD, typically-developing; WGDT, within-channel (2–2 kHz) gap detection threshold.

Table 2 shows that the AGDT scores were the highest (poorest), followed by the WGDT and BBN GDT for both groups of participants. We could also note that the thresholds for the APD group were poorer than those of the TD group in all the GDT measures. Fig. 1 shows scatterplots of individualized absolute ear differences on selected GDT measures categorized between the groups. For the APD group, the Wilcoxon signed-rank test indicated that GDT scores in the right ear were not significantly different from those obtained in the left ear (BBN GDT: z = −0.446; *p* = 0.656; WGDT: z = −0.510; *p* = 0.610; AGDT: z = −1.177; *p* = 0.239). Similarly, for the TD group, the independent samples *t* -test indicated no significant differences across ears on GDT measures (BBN GDT: t (22) = −0.542; *p* = 0.593; WGDT: t (22) = 1.186; *p* = 0.248; AGDT: t(22) = −0.683; *p* = 0.502).

**Fig. 2:**
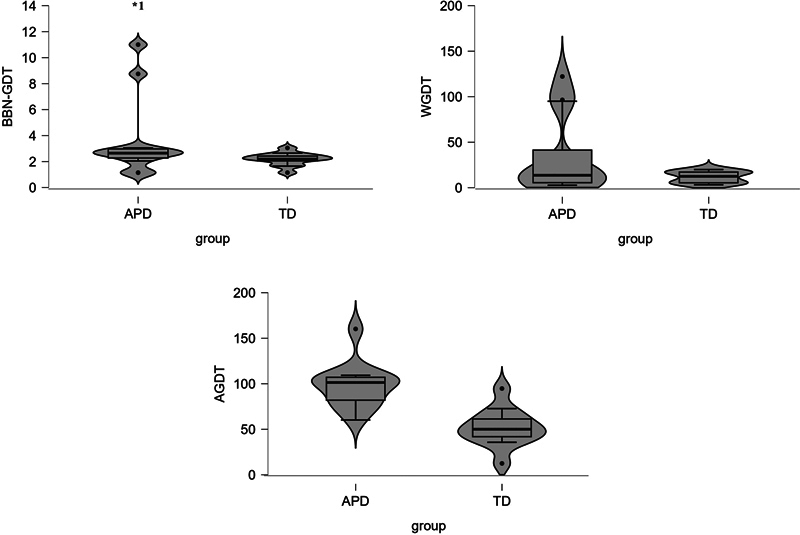
Box plot with violin shade of distribution for the GDTs obtained with various stimuli for TD and APD children. **Abbreviations:** AGDT, across-channel (2–1 kHz) gap detection threshold; APD, auditory processing disorder BBN, broadband noise; GDT, gap detection threshold; TD, typically-developing; WGDT, within-channel (2–2 kHz) gap detection threshold.

Hence, the GDT scores for the right and left ears of each participant were averaged and further analyzed. Fig. 2 shows the box plots for all the temporal measures of the two groups. Fig. 2 also indicates that the two groups performed differently in the AGDT test than in the BBN GDT and WGDT tests. Further, the Mann-Whitney U test was used to assess if the GDT obtained for different stimuli differed between the groups. The results revealed that the AGDT scores were significantly higher in the APD group than in the TD group (U = 7.0; z = −3.753; *p* = 0.000). However, no significant differences were observed in the WGDT values obtained (U = 58.5; z = −0.780; *p* = 0.436) or in the BBN GDT values (U = 40.5; z = −1.827; *p* = 0.068) among children with APD and TD.

## Discussion

The findings of the present study provide valuable insights into the GDTs obtained with different stimuli in two groups of children. Overall, the results indicated that children diagnosed with APD demonstrate deviant temporal processing on GDT measures with any variant of the stimulus used in the current study. However, only the GDT obtained with across-channel narrowband noises demonstrated significant differences between the two groups. These results were analogous to those obtained by Phillips et al. 14 Furthermore, the results provide precise BBN GDT and AGDT values in both normal-hearing children and children with APD using an adaptive measurement procedure. Despite several differences in the stimuli, the results of the present study have also indicated an average of 51.75 ms of AGDT in children with normal hearing, which was very close to the best gap duration of 53 ms reported by Phillips et al. 14 The results of the present study also indicate a large variability in the AGDT scores (SD > 20 ms) in both groups. However, an average AGDT of 98.1 ms was obtained with across-channel stimulus in the APD children. Such AGDTs are higher than the best gap duration for children at risk of APD of 80 ms as reported by Phillips et al. 14 using fixed gap identification tasks.

The results of the current study also reveal an average BBN GDT of 2.19 ms, notably lower compared to similar investigations conducted previously. For instance, Ismaail et al. 26 reported higher average GDTs in children of the same age group, with values ranging from 4.85 ms for the right ear to 5 ms for the left ear. Similarly, Jung and Lee 27 reported an average GDT of 5.4 milliseconds, and Amaral and Colella-Santos, 28 of 4.7 ms, while Shinn et al. 29 reported a range of 4 to 5.8 ms. Most of the previous research, including the aforementioned studies, primarily employed fixed interval gaps in noise to establish gap thresholds. In contrast, in the present study, we used the adaptive staircase procedure combined with an alternative forced-choice method, enabling a more dynamic and precise assessment of auditory temporal acuity perception.

Although the absolute difference between the average BBN GDTs reported in previous studies (ranging from 4.5–6.0 ms) and in the present study (2.19 ms) may appear minimal, it holds significant clinical relevance. In the current study, children with APD also demonstrated an average GDT of 5.25 ms using BBN. This underscores the importance of accurate and sensitive measures of auditory processing abilities, particularly in pediatric populations with suspected auditory deficits. The superior performance observed in the present study suggests that adaptive procedures hold advantages over fixed-interval methods. Moreover, such differences highlight the need for updated normative data while incorporating adaptive testing protocols. Such updated norms would enhance the clinical accuracy of auditory assessments, especially in pediatric populations, in whom early detection and intervention are critical for optimal outcomes.

Further, the findings of the current study revealed notable differences in GDT among TD children across different types of stimuli. Specifically, the average AGDT was 51.75 ms, and the average WGDT, of 11.75 ms. The results of the present study on WGDT agree with those reported by Alhaidary et al. 6 in young adults. However, the young adults who participated in their study 6 presented lower AGDT scores (an average of 29.71 ms) using similar adaptive procedures and 2 to 1 kHz markers. These findings suggest the possibility of developmental changes in AGDT abilities between children and adults, which may be influenced by the maturation of different auditory processing pathways.

Additionally, based on the data obtained, subjects with APD presented good BBN GDT in spite of the deviated performance in the AGDT. Table 3 shows the individual data of BBN GDT, AGDT and WGDT for the APD group. The shaded area represents children with poor scores (mean ± SD of the TD children). Based on Table 3 , it can be noted that the sensitivity of the AGDT measures (75%: 9 out of 12 individuals demonstrating difficulty) is comparatively higher than that of the BBN GDT (∼ 50%) or the WGDT (∼ 33.33%). Thus, the results indicate possible stimulus-specific deficits in children with APD rather than a generalized auditory processing impairment. The elevated AGDTs suggest deficits in temporal processing abilities among different spectral cues might not be processed accurately in this population. Moreover, the higher SD in the APD group suggests heterogeneity within the population, underscoring the need for individualized assessment and intervention strategies tailored to the specific needs of each child. The larger variations observed in AGDT scores both in normal-hearing and APD children indicate that attention has played a significant role in the performance with adaptive measures. 17 Furthermore, Fig. 1 also shows that the absolute difference among ears is comparatively higher in the APD group than in TD group. Thus, ear differences across different stimuli on GDT measures need to be further explored.

**Table 3:** Individual temporal resolution test profiles of the children in the APD group

No.	Age (years)	Sex	BBN-GDT*		WGDT*		AGDT*	
			R	L	R	L	R	L
Subject 1	12	M	1.8	2.3	6.0	1.0	67.5	85.5
Subject 2	12	M	2.8	3.3	2.5	3.0	72.5	126.5
Subject 3	12	F	2.5	2.5	4.0	6.0	113.5	67.5
Subject 4	11	F	2.5	2.8	20.0	27.0	53.5	67.0
Subject 5	11	F	3.3	2.0	9.0	19.6	95.0	118.0
Subject 6	11	F	2.8	2.5	2.0	24.5	104.0	114.5
Subject 7	10	F	28.3	16.8	121.5	71.5	83.0	67.0
Subject 8	10	M	3.3	2.5	7.5	37.0	67.0	100.5
Subject 9	11	M	12.5	5.0	132.5	112.0	83.0	134.5
Subject 10	10	M	1.3	1.0	13.0	9.0	215.5	105.0
Subject 11	10	F	0.8	1.5	6.5	5.0	79.5	128.0
Subject 12	12	M	5.0	17.0	92.5	97.5	102.5	104.0
Criteria used			**> 2.7**	**> 2.8**	**> 20.7**	**> 16.5**	**> 70.1**	**> 77.6**

Abbreviations: AGDT, across-channel gap detection threshold; APD, auditory processing disorder; BBN-GDT, gap detection thresholds obtained with broadband noise markers; F, female; L, left ear; M, male; R, right ear; WGDT, within-channel gap detection threshold (2 kHz).

**Note:** The shaded fields indicate that obtained score is higher than established criteria (mean + 1 standard deviation).

In conclusion, the present study provides valuable insights into the auditory processing difficulties experienced by children diagnosed APD and the efficacy of adaptive procedures in assessing auditory temporal acuity perception. These findings highlight the importance of early identification and intervention to help children develop effective auditory processing skills and advocate for further research to explore the underlying mechanisms of APD and develop targeted interventions to improve the outcomes for affected individuals.

## Conclusion

The results show that, among the three different stimuli presented for GDTs, the AGDT presented higher differences in the performance of children with APD compared to their peer group. Thus, based on the results, we conclude that, compared to other stimuli, an AGDT test would be a better diagnostic test of temporal resolution to identify and assess children with APD.

## Limitations and Suggestions

The present study involved a limited number of participants due to the lack of availability of subjects regarding the selection criteria; moreover, all the measures were performed in a semicontrolled school environment. Future studies may consider involving a high number of participants to generalize the effects observed in the present study. Technically, the authors would encourage the use of higher intertrial intervals when testing children of young age and also the involvement of three alternative forced-choice methods or the standard two alternative forced-choice methods for gap detection measures instead of two alternative forced-choice methods to reduce the higher probability of false-hit rate.
